# Three Bianthraquinone Derivatives from the Mangrove Endophytic Fungus *Alternaria* sp. ZJ9-6B from the South China Sea

**DOI:** 10.3390/md9050832

**Published:** 2011-05-12

**Authors:** Cai-Huan Huang, Jia-Hui Pan, Bin Chen, Miao Yu, Hong-Bo Huang, Xun Zhu, Yong-Jun Lu, Zhi-Gang She, Yong-Cheng Lin

**Affiliations:** 1 School of Chemistry and Chemical Engineering, Sun Yat-sen University, Guangzhou 510275, China; E-Mails: caihuan2@sina.com.cn (C.-H.H.); 609200397@qq.com (J.-H.P.); 32538503@qq.com (B.C.); syhhb007@163.com (H.-B.H.); cesshzhg@mail.sysu.edu.cn (Z.-G.S.); 2 School of Science and Engineering, Jinan University, Guangzhou 510632, China; E-Mail: 63160110@qq.com; 3 Guangdong Province Key Laboratory of Functional Molecules in Oceanic Microorganism (Sun Yat-sen University), Bureau of Education of Guangdong, Guangzhou 510080, China; E-Mail: zhuxun333@gmail.com; 4 School of Life Sciences, Sun Yat-sen University, Guangzhou 510275, China; E-Mail: luyj@mail.sysu.edu.cn

**Keywords:** endophytic fungus, *Alternaria* sp., bianthraquinone, alterporriol, cytotoxicity

## Abstract

Three new bianthraquinone derivatives, alterporriol K (**1**), L (**2**) and M (**3**), along with six known compounds were obtained from extracts of the endophytic fungus *Alternaria* sp. ZJ9-6B, isolated from the mangrove *Aegiceras corniculatum* collected in the South China Sea. Their structures were elucidated by one- and two-dimensional NMR spectroscopy, MS data analysis and circular dichroism measurements. Compounds **1**, **2** and **3** were first isolated alterporriols with a C-2–C-2′ linkage. The crystallographic data of tetrahydroaltersolanol B (**7**) was reported for the first time. In the primary bioassays, alterporriol K and L exhibited moderate cytotoxic activity towards MDA-MB-435 and MCF-7 cells with IC_50_ values ranging from 13.1 to 29.1 μM.

## 1. Introduction

A wide variety of anthraquinone derivatives isolated from plants, animals and marine fungi have served as candidates for various therapeutic uses [1–3]. Anthraquinones inhibit the proliferation of human breast, colon and lung cancer cells [4]. They also displayed inhibitory ability towards protein kinase, NADH oxidase, quinone reductase and calmodulin [5–8]. Several laboratories have investigated anthraquinones as antibacterial agents [9].

The alterporriol family of bianthraquinone derivatives were first reported from *Alternaria porri* by Suemitsu *et al.* in 1984 [10]. Over the last 27 years, nine additional alterporriols have been reported from fungi. All the alterporriols except alterporriols G-J were described from *Alternaria* sp. [5,11–13]. In terms of the underlying monomers, alterporriols can occur as either homodimers or heterodimers. With regard to the coupling positions of the monomers, alterporriols A, B, D, E, I and J feature a C-5–C-5′ linkage, alterporriol C shows a C-1–C-7′ connection, and G and H possess a C-7–C-5′ linkage [5,11–13].

**Figure 1.**
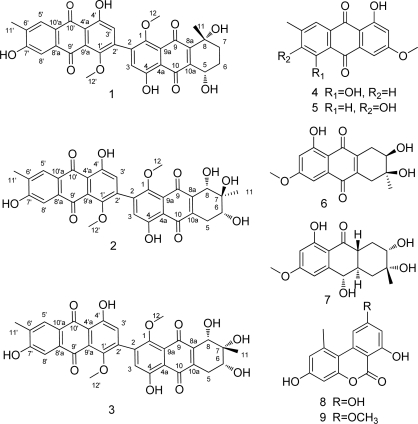
Structures of **1**–**9** isolated from *Alternaria* sp. ZJ9-6B.

As part of our ongoing program to search for new bioactive natural products from the South China Sea [14–16], an endophytic fungus *Alternaria* sp. ZJ9-6B has been isolated from the fruit of the marine mangrove *Aegiceras corniculatum* in Zhanjiang, Guangdong, China. Chemical investigation of this fungus led to the isolation of nine metabolites, including three new anthraquinone derivatives **1**–**3** and six known compounds **4**–**9** (Figure 1). It is interesting that compounds **1**–**3** all possess dimeric structures with a C-2–C-2′ linkage. In this report, we describe the isolation, structural elucidation and biological activity of these new metabolites.

## 2. Results and Discussion

The methanol extract of the dried mycelium was subjected to a combination of column chromatography on silica gel, Sephadex LH-20 and C_18_ reversed phase silica gel.

**Figure 2.**
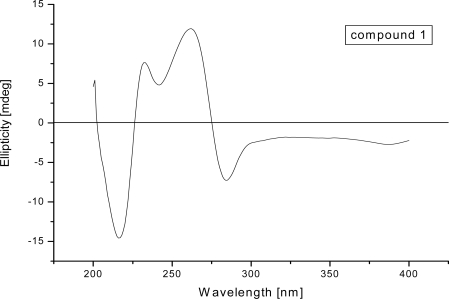
CD Spectra of **1**. Recorded in MeOH at amibient temperature.

**Figure 3.**
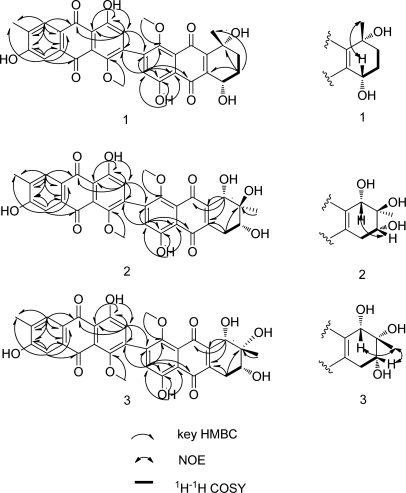
Key HMBC, NOE and ^1^H-^1^H COSY correlations of **1**–**3**.

Compound **1** was isolated as a red amorphous powder. HR-EIMS at *m/z* = 586.1471 [M]^+^ indicated the molecular formula C_32_H_26_O_11_ (calcd. for C_32_H_26_O_11_, 586.1470). Compound **1** exhibited strong optical rotation ${\left[\text{α}\right]}_{\text{D}}^{20}$ (*c* = 1.0, MeOH) which indicated the possibility of an asymmetric centre and/or axial chirality (Figure 2). The IR spectrum (KBr) exhibited a weak shoulder at 1652 cm^−1^ and an intense band at 1638 cm^−1^ for carbonyl groups. The UV spectrum displayed bands at 224, 280 and 437 nm, suggesting a quinonoid chromophore. The ^1^H NMR spectrum (Table 1) showed a pair of chelated hydroxyl resonances (δ_H_ = 13.61 and 13.15 ppm), four aromatic protons (δ_H_ = 7.67, 7.55, 6.92 and 6.88 ppm), two methoxyl protons (δ_H_ = 3.68 and 3.66 ppm), two singlet methyls (δ_H_ = 2.18 and 1.07 ppm), two methylene protons (δ_H_ = 2.53 and 2.72 ppm, δ_H_ = 2.20 and 2.34 ppm), and oxygenated methine (δ_H_ = 3.51 ppm). The ^13^C NMR spectrum displayed four carbonyl signals (δ_C_ = 183.6, 187.8, 181.1 and 186.7 ppm), twenty signs of aromatic carbons, one quaternary carbon (δ_C_ = 69.0 ppm), one methine (δ_C_ = 70.1 ppm) and two methylenes (δ_C_ = 29.1 and 36.1 ppm). These data implied that compound **1** possessed a bianthranquinone scaffold, including an anthraquinone unit and a tetrahydroanthraquinone unit (Figure 1) [5,13]. The unsubstituted carbons for two aromatic rings of the anthraquinone unit were located at C-8′ (δ_C_ = 130.3 ppm; δ_H_ = 7.67 ppm, d, *J* = 0.8 Hz), C-5′ (δ_C_ = 110.5 ppm; δ_H_ = 7.55 ppm, d, *J* = 0.8 Hz) and C-3′ (δ_C_ = 103.8 ppm; δ_H_ = 6.921 ppm, s) by the HMBC correlations (Figure 3). In the tetrahydroanthraquinone unit, one aromatic proton at H-3 (δ_H_ = 6.88 ppm, s) and the protons in the alicyclic ring, including one oxygenated methine H-5 (δ_H_ = 3.51 ppm, ddd, *J* = 5.4, 5.5, 12.5 Hz) and two methylene protons H-6 (δ_H_ = 2.53 and 2.72 ppm) and H-7 (δ_H_ = 2.20 and 2.34 ppm) were observed.

A contiguous sequence of coupled signals from H-5 to H-7 in the ^1^H-^1^H COSY spectrum combined with the HMBC correlations from H-5 to C-6, C-7 and C-8a, from H-6 to C-5, C-8, and C-10a, and from H-7 to C-5, C-8, C-8a, C-9 and C-11, established the substructure of the cyclohexene ring (Figure 3).

HMBC correlations from each of 4-OH (δ_H_ = 13.15 ppm) and 4′-OH (δ_H_ = 13.63 ppm) to C-4, C-3, C-4a and to C-4′, C-3′, C-4′a, respectively, indicated their location at C-4 and C-4′ of the bianthraquinone scaffold.

The presence of a bond connecting C-2 and C-2′ was suggested by the absence of two sets of *ortho*-coupled doublets and the presence of two singlets H-3 (δ_H_ = 6.88 ppm, s) and H-3′ (δ_H_ = 6.92 ppm, s). Moreover, HMBC correlations of H-3 with C-1, C-2, C-2′, C-4, C-4a and C-10, and of H-3′ with C-1′, C-2, C-2′, C-4′, C-4′a and C-10′, respectively (Figure 3) provided evidence for C-2–C-2′ linkage of **1**. The HMBC spectrum showed correlations of the methoxyl proton (δ_H_ = 3.66 ppm, s, 3H, H-12) with C-1 and C-3. Likewise, correlations were observed from methoxyl proton (δ_H_ = 3.69 ppm, s, 3H, H-12′) to C-1′. These allowed us to assign two methoxyl groups to C-1 and C-1′.

The relative configuration of the chiral centers of C-5 and C-8 were deduced by 2D ^1^H-^1^H NOESY experiments (Figure 3) and the analysis of ^1^H-^1^H coupling constants (Table 1). A NOESY correlation between CH_3_-11 (δ_H_ = 1.07 ppm, s) and H-5 suggested that they were on the same side of the cyclohexene ring. The axial position of H-5 was confirmed by coupling constant *J*_H-5,H-6a_ = 12.5 Hz, indicating CH_3_-11 to also have an axial orientation. Therefore, compound **1** was determined as (5S*,8R*)-4,4′,5,7′,8-pentahydroxy-1,1′-dimethoxy-6′,8-dimethyl-5,6,7,8-tetrahydro-[2,2′-bianthracene]-9,9′,10,10′-tetraone. We propose the trivial name alterporriol K.

Compounds **2** and **3** were obtained as a mixture after separating with Sephadex LH-20 chromatography. Upon HPLC analysis, the mixture exhibited two partially overlapped peaks (area ratio *ca.* 4:1). Then compounds **2** and **3** were isolated with re-separation by preparative HPLC, respectively. Compound **2** was a red amorphous powder, ${\left[\alpha \right]}_{\text{D}}^{25}\;=\;+30$ (*c* = 1.0, MeOH). The HR-ESI-TOF-MS exhibited a peak at *m/z* = 601.1340 [M – H]^−^ indicating a molecular formula of C_32_H_26_O_12_ (calcd. for C_32_H_25_O_12_, 601.1346). Comparison of the ^1^H and ^13^C NMR spectral data of **2** (Table 2) with that of **1** showed a close structural relationship between both compounds, except for the presence of an additional oxymethine group proton (δ_H_ = 4.03, d, *J* = 6.7 Hz, H-8) and an additional hydroxyl group signal (δ_H_ = 2.17 ppm, s, 6-OH) and the absence of the methylene signals corresponding to H-7 in the ^1^H NMR spectrum of **2**. The substructure of the cyclohexene ring was established by HMBC correlations from H-8 to C-6, C-7, C-8a, C-9, C-10a and C-11 and ^1^H-^1^H COSY correlations between H-5 and H-6 (Figure 3). NOE difference and analysis of ^1^H-^1^H coupling constants (Table 2) enabled the relative of configuration of **2** to be deduced. In the NOE experiment, when the methyl signal CH_3_-11 (δ_H_ = 1.16, s) was irradiated, no enhancement of signals H-6 and H-8 was observed. Meanwhile, irradiation of H-8 caused an enhancement of H-6. These data suggested that CH_3_-11 with H-6 and H-8 was *trans*-configuration in the cyclohexene ring (Figure 3). A large coupling constant for H-5a/H-6 (*J*_H-5a,H-6_ = 10.0 Hz) indicated an axial location for H-6, likewise suggesting an axial location for H-8. Thus, the relative configuration at C-6, C-7, and C-8 was 6S*, 7R* and 8R*. Compound **2** was finally defined as (6S*,7R*,8R*)-4,4′,6,7,7′,8-hexahydroxy-1,1′-dimethoxy-6′,7-dimethyl-5,6,7,8-tetrahydro-[2,2′-bianthracene]-9,9′,10,10′-tetraone, named as alterporriol L.

Compound **3**, obtained as a red amorphous power, ${\left[\alpha \right]}_{\text{D}}^{25}\;=\;-90$ (*c* = 1.0, MeOH), had the molecular formula C_32_H_26_O_12_, as determined by HR-ESI-TOF-MS at *m/z* = 601.1340 [M – H]^−^ (calcd. for C_32_H_25_O_12_, 601.1346). The MS analysis revealed **3** to be an isomer of **2**. The UV and IR absorptions, ^1^H and ^13^C NMR data, HMBC and ^1^H-^1^H COSY correlations (Figure 3 and Table 2) of **3** were almost identical with those of **2**, suggesting the two compounds to have identical carbon skeletons. However, in contrast with 2 in the NOE experiment of 3, irradiation of the methyl signal CH_3_-11 (δ_H_ = 1.16 ppm, s) resulted in obvious enhancements of the signals for both H-6 and H-8. The coupling constant for H-5a/H-6 was measured to be *J*_H-5a,H-6_ = 9.9 Hz suggesting axial locations for H-6 and, by extension, H-8. These data suggested that 2 and 3 are epimers at C-7. Therefore compound 3 was elucidated as (6S*,7S*,8R*)-4,4′,6,7,7′,8-hexahydroxy-1,1′-dimethoxy-6′,7-dimethyl-5,6,7,8-tetrahydro-[2,2′-bianthracene]-9,9′,10,10′-tetraone, named alterporriol M.

**Figure 4.**
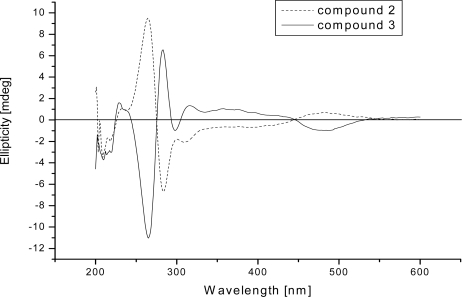
CD Spectra of **2** and **3**. Recorded in MeOH at amibient temperature.

An interesting feature of the isolated bianthquinones is their optical activity. *Ab initio* calculations of CD spectra for several phenylanthraquinones with chiral axes have recently been reported [17,18]. Specific optical rotation of compounds **2** and **3** showed ${\left[\text{α}\right]}_{\text{D}}^{20}\;+60$ and −30, respectively. CD spectra of **2** and **3** recorded in methanol showed a near quasi-mirror image pattern (Figure 4). In the case of **2**, the CD spectrum consists of a small positive band at 480 nm, followed by two moderate negative bands in the 300–400 nm regions, and a stronger negative band centered at 220 nm, then a stronger positive band at 264 nm. For **3,** the sequence of bands is similar but their signs are inverted. The CD spectra of **2** and **3** reflect contributions from a chiral axis and three chiral centers. The chiral axis occurring within the chromophore is expected to dominate the observed CD spectrum [18,19] (Figure 4).The chiral center C-7 is expected to have a much smaller contribution to the observed CD spectrum. With these results, compounds **2** and **3** were deduced as two diastereomers and atropisomers [20].

**Figure 5.**
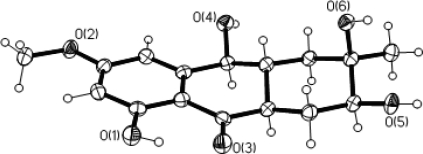
X-ray crystal structure of **7**.

The known compounds were identified as physcion (**4**), marcrospin (**5**), dactylariol (**6**), tetrahydroaltersolanol B (**7**), alternariol (AOH) (**8**) and alternariol methyl ether (AME) (**9**) by spectral analyses and comparison with reported literature data, respectively [21–24]. The structure of **7** was confirmed by X-ray diffraction analysis (Figure 5) and its crystallographic data was reported for the first time.

Compounds **1** and **2** were evaluated for their cytotoxicity against human breast cancer cell lines MDA-MB-435 and MCF-7 by MTT assay. Compound **1** had an IC_50_ value of 26.97 μM against MDA-MB-435 and 29.11 μM against MCF-7 cells, respectively. Compound **2** showed activities against MDA-MB-435 (IC_50_ = 13.11 μM) and MCF-7 (IC_50_ = 20.04 μM). No biological studies were performed for compound **3** due to the limited yield.

## 3. Experimental Section

### 3.1. General

Column chromatography (CC) was performed using silica gel (200–300 mesh, Qingdao marine Chemical). The HPLC system consisted of a Waters 2010 series. A mini ODS column (250 × 10 mm, 10 μm particle size) was used. Melting points were determined on an X-4 micro-melting point apparatus and were uncorrected. Circular dichroism was measured on a Schmidt Haensch Polartronic HH W5 polarimeter and was uncorrected. UV spectra were measured on a Shimadzu UV-2501 PC spectrophotometer. IR spectra were measured on a Bruker EQUINOX55 spectrophotometer. ^1^H and ^13^C NMR data were recorded on a Varian Inova 500 NB and a Burker AVANCE 400 spectrometer, respectively (TMS as internal standard). EIMS were on a Thermo DSQ EI-mass spectrometer. LC/MS data were acquired using an Applied Biosystems/MDS Sciex and ESI source. HR-EIMS were measured on a Thermo MAT95XP High Resolution mass spectrometer. HR-ESIMS were measured on a Schimadzu LCMS-IT-TOF.

### 3.2. Strain Isolation, Taxonomic Classification and Endophyte Fermentation

The fungus *Alternaria* sp. ZJ9-6B was isolated from the fruit of a mangrove tree *Aegiceras Corniculatum* collected in Zhanjiang Mangrove, Guangdong province, P.R. China, in 2008. A voucher specimen (registration number: ZJ9-6B) has been deposited in the Natural Products Laboratory and the Department of Applied Chemistry, Sun Yat-sen University, China. It was identified according to a molecular biological protocol by DNA amplification and sequencing of the ITS region as described previously with an ITS sequence GenBank ID: HM 754629. The fungal strain was cultivated in potato dextrose broth (PDB) medium (20 g of dextrose and 3 g of crude sea salt in 1 L of potato infusion). Starter cultures were maintained on cornmeal seawater agar. Plugs of agar supporting mycelia growth were cut and transferred aseptically into a 500 mL Erlenmeyer flask containing 250 mL of liquid medium, and incubated at 28 °C on a rotary shaker for 5–7 days. The mycelium was aseptically transferred into 1000 mL Erlenmeyer flasks containing 500 mL PDB medium and incubated at 28 ± 1 °C for 30 days under stationary conditions.

### 3.3. Extraction and Separation of Metabolites

The cultures (200 L) were separated into mycelium and filtrate. The dried mycelium (724 g) was extracted with methanol (6 L × 4) to give 151.6 g of a crude extract. The crude extract was subjected to silica gel CC using gradient elution with petroleum-ether (PE) and ethyl acetate (EA) mixture (v/v, 95:5–0:100) to give six fractions (A–F). Fraction C (27.1 g) was purified by silica CC with a PE-EA mixture (v/v, 85/15, 4 L) to give three subfractions C-1 (8.7 g), C-2 (5.1 g) and C-3 (3.5 g). These three subfractions were further purified by silica gel CC with a PE-EA mixture to give **4** (161 mg), **5** (67 mg), **8** (19 mg) and **9** (28 mg). Fraction D (13.5 g) was isolated by silica gel CC with PE-EA (v/v, 75:25, 3 L) to give two subfractions D-1 (2.7 g) and D-2 (3.6 g). These two subfractions D-1 and D-2 were further purified by Sephadex LH-20 gel CC with CHCl_3_-MeOH (65:35, v/v) as a mobile phase and by preparative HPLC with an ODS column (10 × 250 mm), eluting with MeOH-H_2_O (68:32, v/v) to give **1** (11 mg), **2** (18.4 mg), **3** (3 mg), **6** (13 mg) and **7** (21 mg).

**Alterporriol K:** Red powder. ${\left[\alpha \right]}_{\text{D}}^{25}\;=\;+690$ (*c* = 1.0, MeOH). UV (MeOH): λ_max_ (log ε) = 279.80 (1.52), 223.80 (1.96) nm. IR (KBr): ν_max_ = 3448, 2966, 2855, 1652, 1638, 1595, 1464, 1430, 1388, 1289, 1208, 1111, 1072, 973, 933, 842, 621 cm^−1^. For ^1^H, ^13^C and 2D NMR spectroscopic data, see Table 1. HR-EIMS: calcd. for C_32_H_26_O_10_, 586.1470; found *m/z* = 586.1471 [M]^+^.

**Alterporriol L:** Red powder. ${\left[\alpha \right]}_{\text{D}}^{25}\;=\;+30$ (*c* = 1.0, MeOH). UV (MeOH): λ_max_ (log ε) = 280.20 (1.24), 225.40 (1.61) nm. IR (KBr): ν_max_ = 3440, 2926, 2857, 1592, 1462, 1430, 1389, 1288, 1208, 1109, 1070, 974, 927, 846, 793, 605 cm^−1^. For ^1^H, ^13^C and 2D NMR spectroscopic data, see Table 2. HR-ESIMS: calcd. for C_32_H_25_O_12_, 601.1346; found *m/z* = 601.1340 [M – H]^−^.

**Alterporriol M:** Red powder. ${\left[\alpha \right]}_{\text{D}}^{25}\;=\;-90$ (*c* = 1.0, MeOH). UV (MeOH): λ_max_ (log ε) = 279.00 (1.33), 225.60 (1.77) nm. IR (KBr): ν_max_ = 3450, 2928, 2027, 1638, 1463, 1389, 1280, 1207, 1111, 1046, 977, 929, 858, 618 cm^−1^. For ^1^H, ^13^C and 2D NMR spectroscopic data, see Table 2. ESIMS *m/z* = 601.1 [M – H]^−^. HR-ESIMS calcd. for C_32_H_25_O_12_, 601.1346; found *m/z* = 601.1340 [M – H]^−^.

**Crystallographic data of 7:** The X-ray diffraction data for **7** (Figure 5) was measured on an Xcalibur Nova 1000 CCD diffractometer (Mo Kα-radiation, graphite monochromator). Solvent for crystallization: methanol; Wavelength: 0.71073 Å; Temperature: 173 K; Empirical formula: C_16_H_20_O_6_; Crystal system: monoclinic; Space group C2 with *a* = 37.306(6) Å, *b* = 5.8251(10) Å, *c* = 7.9087(13) Å, α = 90.00°, β = 102.210(3)°, γ = 90.00°; *V* = 1679.8(5) Å^3^; Density: 1.397 g/cm^3^; *Z* = 2, *F* (000) = 756.0; Goodness-of-fit on *F*^2^: 1.087; *R* Indices (all data): *R*_1_ = 0.0399, *wR*_2_ = 0.1208. CCDC-751689 contains the supplementary crystallographic data for this paper. These data can be obtained free of charge via www.ccdc.cam.ac.uk/conts/retrieving.html (or from the CCDC, 12 Union Road, Cambridge CB2 1EZ, UK; Fax: +44-1223-336033; E-Mail: deposit@ccdc.cam.ac.uk).

### 3.4. Cytotoxic Assays

The cytotoxicity of compounds **1** and **2** were evaluated using human breast cancer cell lines MDA-MB-435 and MCF-7 by MTT assay. Cell viability was measured using the CellTiter 96 aqueous nonradioactive cell proliferation assay. Results were expressed as the mean value of triplicate data points.

## 4. Conclusions

*Alternaria* sp. ZJ9-6B is a prolific producer of bioactive metabolites. Nine compounds have been isolated from this fungal strain, including three new alterporriols. These three alterporriols all possess dimeric structures with a C-2–C-2′ linkage. In the primary bioassay, compounds **1** and **2** showed moderate cytotoxic activity against human breast cancer cell lines.

## Supplementary Data



## Figures and Tables

**Table 1.** NMR spectroscopic data (DMSO-*d_6_*) of **1**^a,b^.

**Atom**	**δ_C_ (ppm)**	**δ_H_ (ppm) (multiplicity, *J*(Hz))**	**COSY^b^**	**HMBC^b^**	**NOESY**
1	164.1				
2	123.3				
3	103.7	6.88 (s)		C-1, 2, 2′, 4, 4a, 10	
4	163.8				
4a	108.6				
5	70.1	3.51 (ddd, 5.5, 5.5, 12.5)	H-6a, 6b, 5-OH	C-6, 7, 8a, 11	H-11
6	29.1	a: 2.53 (m)	H-5, 7a, 7b	C-5, 8, 10a	H-11
		b: 2.72 (m)	H-5, 6a, 7a, 7b	C-5, 8, 10a, 11	
7	36.1	a: 2.31 (m)	H-6a, 6b, 7b	C-5, 8, 8a, 11	
		b: 2.35 (m)	H-6a, 6b, 7a	C-5, 8, 8a, 11	H-11
8	69.0				
8a	141.6				
9	183.6				
9a	128.9				
10	187.8				
10a	143.2				
11	25.2	1.07 (s)		C-5, 7, 8	
12	56.7	3.66 (s)		C-1, 3	
4-OH		13.15 (s)		C-3, 4, 4a	
5-OH		4.69 (d, 5.5)	H-5	C-5, 6, 8	
8-OH		4.30 (s)		C-5, 7, 8, 11	
1′	164.7				
2′	122.5				
3′	103.8	6.92 (s)		C-2, 2′, 4′, 4′a, 10′	
4′	165.0				
4′a	110.0				
5′	110.5	7.55 (d, 0.8)		C-6′, 7′, 8′a, 10′, 10′a, 11′	
6′	125.2				
7′	161.3				
8′	130.3	7.67 (d, 0.8)		C-7′, 8′a, 9′, 10′a, 11′	
8′a	132.4				
9′	181.1				
9′a	130.7				
10′	186.7				
10′a	132.2				
11′	16.1	2.18 (s)		C-5′, 7′, 8′	
12′	56.8	3.69 (s)		C-1′	
4′-OH		13.63 (s)		C-3′, 4′, 4′a	
7′-OH		8.11 (s)			

a Measured at 500 MHz (for ^1^H) and 125 MHz (for ^13^C);

b For the HMBC and COSY spectra, see the Supporting Information.

**Table 2.** NMR spectroscopic data (DMSO-*d_6_*) of **2** and **3**^a,b^.

**Actom**	**2**			**3**		
	**δ_C_ (ppm)**	**δ_H_ (ppm) (multiplicity, *J* (Hz))**	**HMBC**	**δ_C_ (ppm)**	δ**_H_ (ppm) (multiplicity, *J* (Hz))**	**HMBC**
1	164.3			164.5		
2	123.2			123.5		
3	103.6	6.90 (s)	C-1, 2, 2′, 4, 4a, 10	103.4	6.92 (s)	C-1, 2, 2′, 4, 4a, 10
4	163.8			163.9		
4a	108.8			108.8		
5	28.8	a: 2.33 (dd, 19.5, 10.0)	C-6, 8a, 10a	28.8	a: 2.33 (dd, 19.3, 9.9)	C-6, 8a, 10a
		b: 2.79 (dd, 19.5, 6.0)	C-6, 7, 8a, 10a		b: 2.78 (dd, 19.3, 5.9)	C-6, 7, 8a, 10, 10a
6	66.7	3.70 ^c^		66.6	3.69 ^c^	
7	71.9			71.9		
8	69.0	4.03 (d, 6.7)	C-6, 7, 9, 8a, 10a	69.0	4.06 (d, 5.7)	
8a	142.6			142.6		
9	183.3			183.1		
9a	128.9			128.3		
10	188.4			188.3		
10a	143.7			143.6		
11	21.8	1.16 (3H, s)	C-6, 7, 8	21.8	1.16 (3H, s)	C-6, 7, 8
12	56.7	3.68 (3H, s)	C-1	56.7	3.68 (3H, s)	C-1
4-OH		13.11 (s)	C-3, 4, 4a		13.13 (s)	C-3, 4, 4a
6-OH		2.17 (s)			2.16 (s)	
7-OH		4.25 (s)	C-7, 8		4.48 (s)	
8-OH		5.42 (d, 6.7)	C-6, 7, 8		5.27 (d, 5.7)	
1′	164.4			164.9		
2′	122.3			122.5		
3′	104.0	6.92(s)	C-2, 2′, 4′, 4′a, 10′	103.5	6.89 (s)	C-1′, 2, 2′, 4′, 4′a,10′
4′	164.9			165.1		
4′a	110.0			110.0		
5′	110.5	7.55 (s)	C-6′, 7′, 8′a, 10′, 10′a, 11′	110.7	7.51 (s)	C-6′, 7′, 8′a, 10′, 10′a, 11′
6′	125.0			125.2		
7′	161.6			162.8		
8′	130.2	7.68 (s)	C-7′, 8′a, 9′, 10′a, 11′	130.1	7.65 (s)	C-7′, 8′a, 9′, 10′a, 11′
8′a	132.4			132.4		
9′	181.1			180.6		
9′a	131.2			130.7		
10′	186.8			186.9		
10′a	132.3			132.4		
11′	16.1	2.19 (s)	C-5′, 6′, 7′, 8′, 10′a	16.3	2.18 (s)	C-5′, 6′, 7′, 8′
12′	56.7	3.68 (s)	C-1′	56.8	3.71 (s)	C-1′
4′-OH		13.61 (s)	C-3′, 4′, 9′a		13.70 (s)	C-3′, 4′, 9′a
7′-OH		7.65 (s)	C-7′, 8′a, 9′, 10′, 11′		7.67 (s)	C-5′, 7′, 8′a, 11′

a Measured at 500 MHz (for 1H) and 125 MHz (for 13C);

b For the HMBC and COSY spectra, see the Supporting Information;

c Overlapped by methoxyl signal.
