# Supplementary material for: Three Bianthraquinone Derivatives from the Mangrove Endophytic Fungus Alternaria sp. ZJ9-6B from the South China Sea
Source: Mar Drugs. 2011 May 12;9(5):832–43. doi: 10.3390/md9050832 (PMC3111185; doi:10.3390/md9050832)
Supplement: Supplementary file 1 [file marinedrugs-09-00832-s001.pdf]

# Three Anthraquinone Derivatives from the Marine Mangrove Endophytic Fungus *Alternaria* sp. ZJ9-6B from the South China Sea

**Cai-huan Huang [1, 2], Jia-hui Pan[1], Bin Chen[1], Miao Yu[2], Hong-bo Huang[1], Xun Zhu[4], Yong-Jun Lu[3], Zhi-gang She[1, 4] and Yong-cheng Lin [1, 4,]***

1 School of Chemistry and Chemical Engineering, Sun Yat-sen University, Guangzhou 510275, China; E-mails: caihuan2@sina.com.cn (C.H.); panjiahui@foxmail.com (J.P.);32538503@qq.com (B.C.); 63160110@qq.com (M.Y.); syhhb007@163.com (H.H.); zhuxun333@gmail.com(X. Z.); luyj@mail.sysu.edu.cn (Y.L.); cesshzhg@mail.sysu.edu.cn (Z.S.).
2 School of Science and Engineering, Jinan University, Guangzhou 510632, China
3 School of life Sciences, Sun Yat-sen University, 510275 Guangzhou, China
4 Guangdong Province Key Laboratory of Functional Molecules in Oceanic Microorganism (Sun Yat-sen University), Bureau of Education of Guangdong, Guangzhou, China
* Author to whom correspondence should be addressed; E-mail: ceslyc@mail.sysu.edu.cn; Tel: +86-20-84039623; Fax: +86-20-84039623

**Description of the Anti-cancer Activity Assay**

Preparation of Compounds **1** and **2**

Compounds **1** and **2** were dissolved in 0.5% dimethylsulphoxide (DMSO) at a concentration of 1 mM as stock solution and diluted according to experimental requirements when used.

Cell culture

Human breast cancer cell lines MCF-7 and MDA-MB-435 were cultured in Dulbecco’s modified Eagle’s medium (DMEM) (Invitrogen, Carlsbad, CA, USA) supplemented with 5% fetal bovine serum (Hyclone, Logan, UT, USA), 2mM L-glutamine, 100 mg·mL$^{-1}$ streptomycin and 100 units·mL$^{-1}$ penicillin (Invitrogen). The cultures were maintained at 37°C in a humidified atmosphere of 5% $CO_2$. Cell Viability assay

Cells were seeded in 96-well flat-bottom plates at a density of $1×10^4$ cells per well and cultured in a humidified incubator for 24 h, followed by exposure to various concentrations of compound 1 and 2 for 48 h respectively. Subsequently, 20 μL of 3-(4,5-dimethylthiazol-2-yl)-2,5-diphenyl tetrazolium bromide (MTT) reagent (Genview, Houston, TX, USA) dissolved in phosphate-buffered saline (PBS) (pH 7.4) at a concentration of 5 mg·mL$^{-1}$ was added to each well, and the cells were incubated for additional 4 h. The MTT-formazan crystals formed were dissolved in 150 μL DMSO (Sangon Biotech, Shanghai, China), and the absorbance was measured at 570 nm with a reference wavelength of 630 nm using a microplate reader. Cell growth inhibition was determined using the following formula according to a previously published method: growth inhibition (%) = (1-OD of treated cells/OD of control cells) ×100% (Moon et al., 2000). The half maximal inhibitory concentration ($IC_{50}$) was calculated by Bliss’s software (Bliss, 1935), and the data were analysed by SPSS. For all tests, the inhibition assays were performed in triplicate.

**Table of contents**

**Figure S1. $^{1}$H spectrum of 1 (**500 MHz, DMSO-$d_6$**).**

**Figure S2. $^{1}$C spectrum of 1 (**125 MHz, DMSO-$d_6$**).**

**Figure S3. HMQC spectrum of 1 (**500/125 MHz, DMSO-$d_6$**).**

**Figure S4. HMBC spectrum of 1 (**500/125 MHz, DMSO-$d_6$**).**

**Figure S5. Expansion of HMBC spectrum of 1 (**500 MHz, DMSO-$d_6$**).**

**Figure S6. $^{1}$H-$^{1}$H COSY spectrum of 1 (**500 MHz, DMSO-$d_6$**).**

**Figure S7. HR-EIMS spectrum of 1.**

**Figure S8. $^{1}$H spectrum of 2 (**500 MHz, DMSO-$d_6$**).**

**Figure S9. $^{1}$C spectrum of 2 (**125 MHz, DMSO-$d_6$**).**

**Figure S10. HMQC spectrum of 2 (**500/125 MHz, DMSO-$d_6$**).**

**Figure S11. HMBC spectrum of 2(**500/125 MHz, DMSO-$d_6$**).**

**Figure S12. $^{1}$H-$^{1}$H COSY spectrum of 2 (**500 MHz, DMSO-$d_6$**).**

**Figure S13. HR-ESIMS spectrum of 2**

**Figure S14. $^{1}$H spectrum of 3 (**500 MHz, DMSO-$d_6$**).**

**Figure S15. $^{1}$C spectrum of 3 (**125 MHz, DMSO-$d_6$**).**

**Figure S16. HMQC spectrum of 3 (**500/125 MHz, DMSO-$d_6$**).**

**Figure S17. HMBC spectrum of 3 (**500/125 MHz, DMSO-$d_6$**).**

**Figure S18. $^{1}$H-$^{1}$H COSY spectrum of 3 (**500 MHz, DMSO-$d_6$**).**

**Figure S19. HR-ESIMS spectrum of 3**

**Figure S1.** $^{1}$**H spectrum of 1 (**500 MHz, DMSO-$d_6$**).**

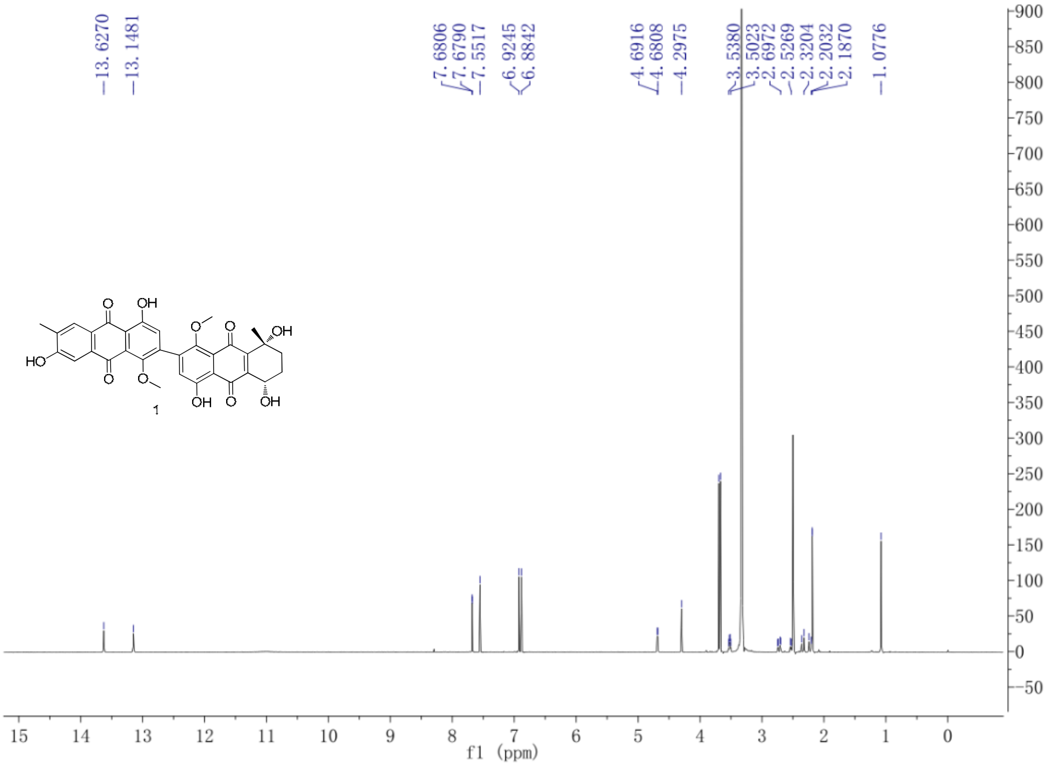

**Figure S2.** $^{13}$**C spectrum of 1 (**125 MHz, DMSO-$d_6$**).**

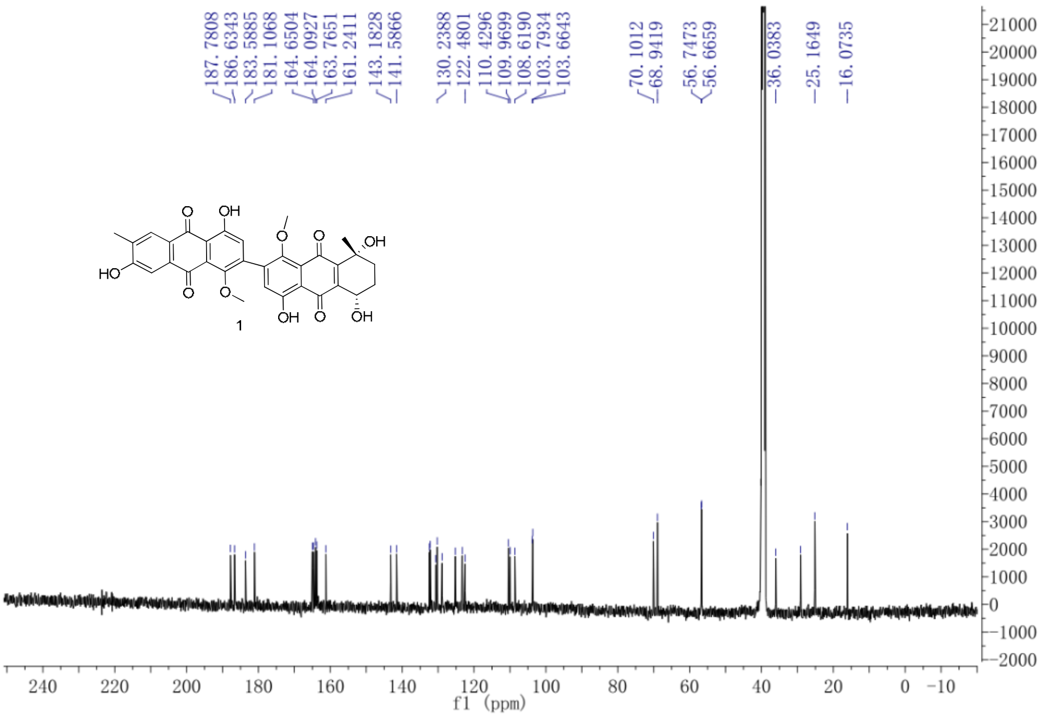

**Figure S3. HMQC spectrum of 1 (**500/125 MHz, DMSO-$d_6$**).**

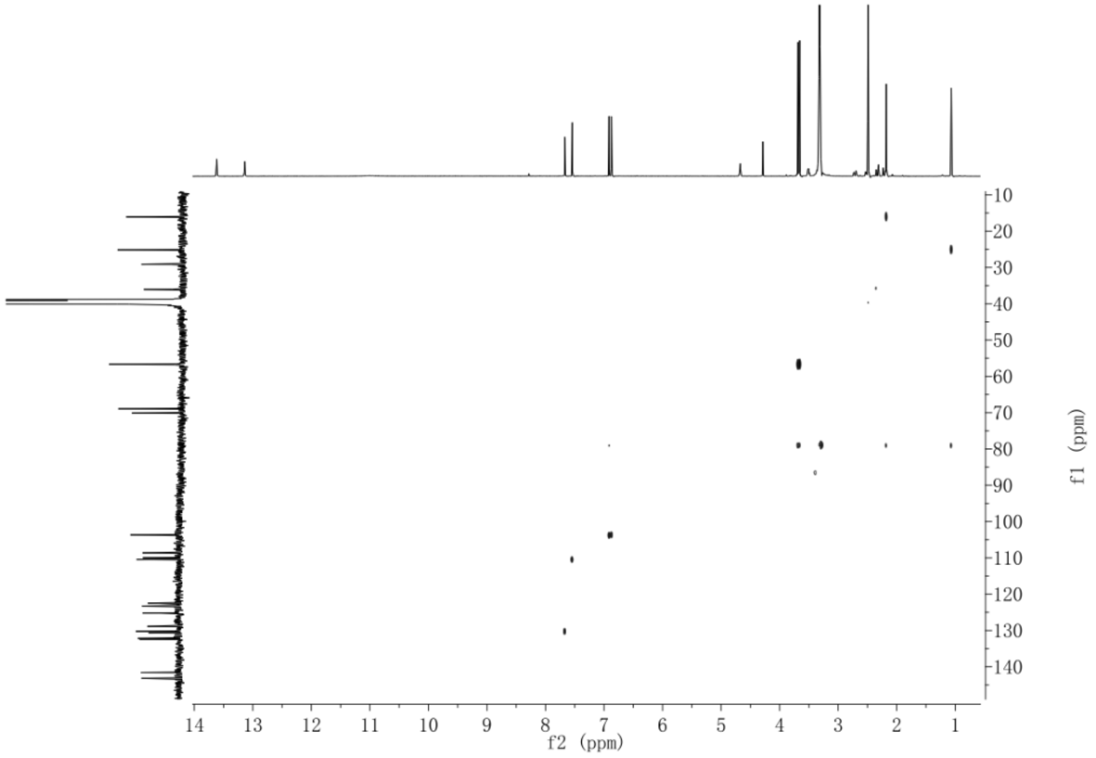


**Figure S4. HMBC spectrum of 1 (**500/125 MHz, DMSO-$d_6$**).**

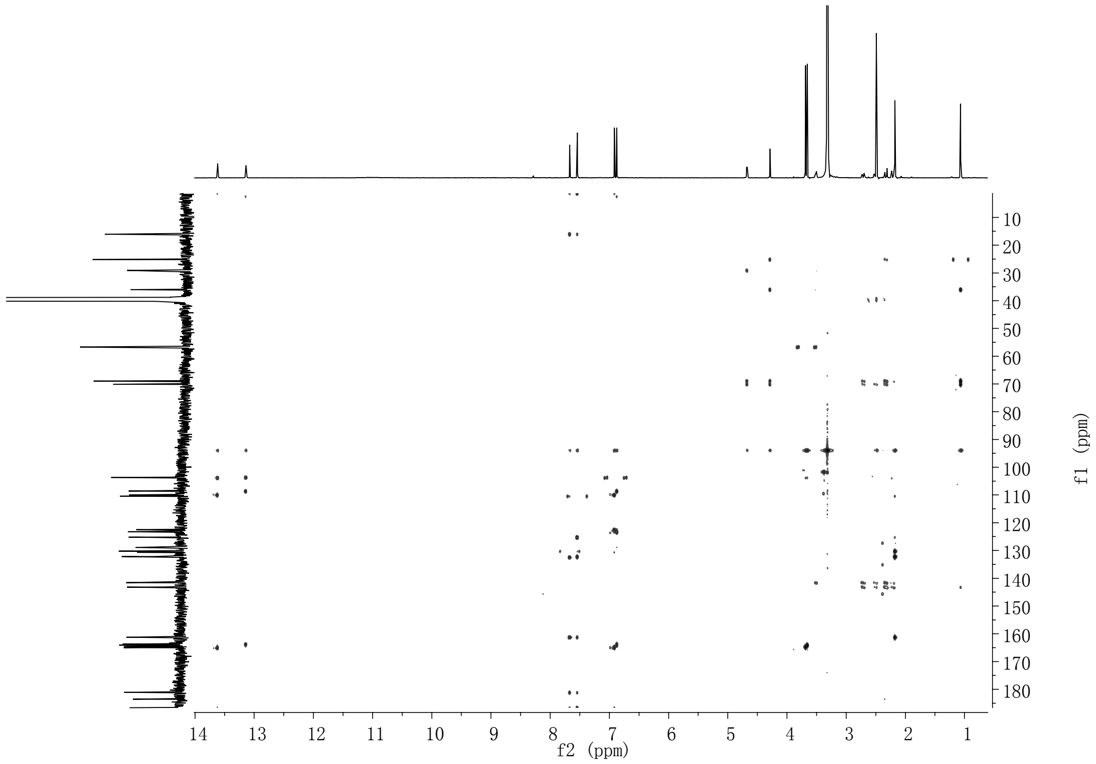

**Figure S5. Expansion of HMBC spectrum of 1 (**500 MHz, DMSO-$d_6$**).**

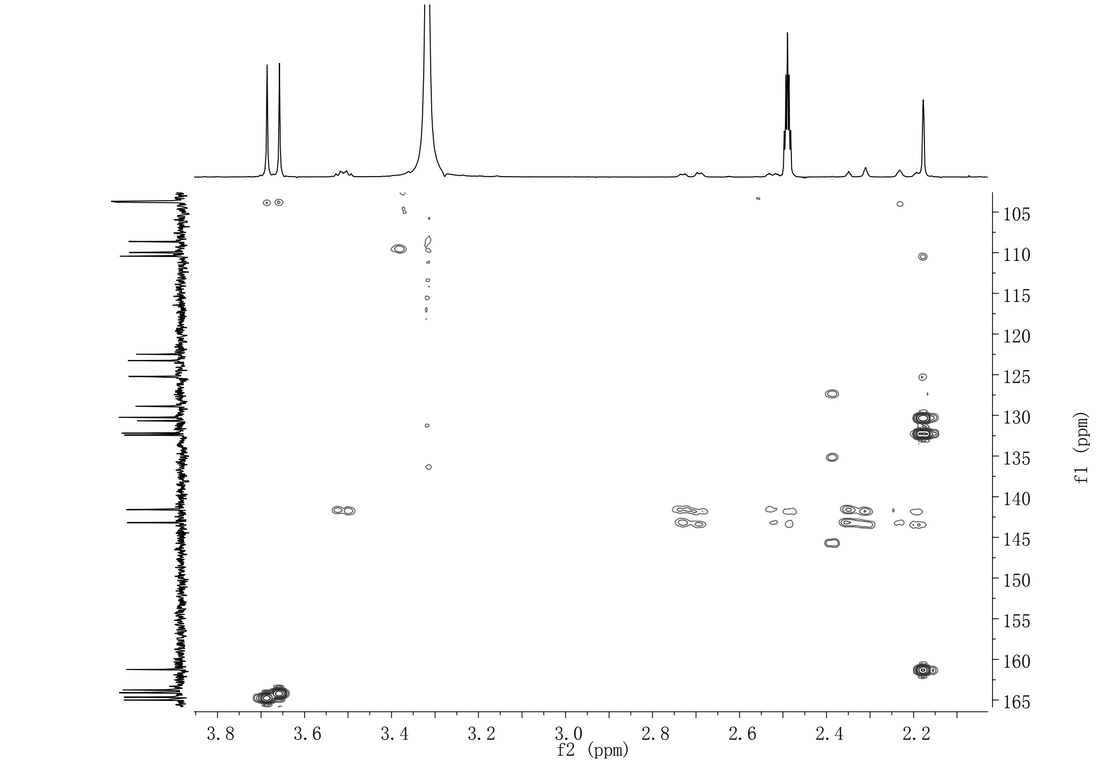


**Figure S6. $^1$H-$^1$H COSY spectrum of 1 (**500 MHz, DMSO-$d_6$**).**

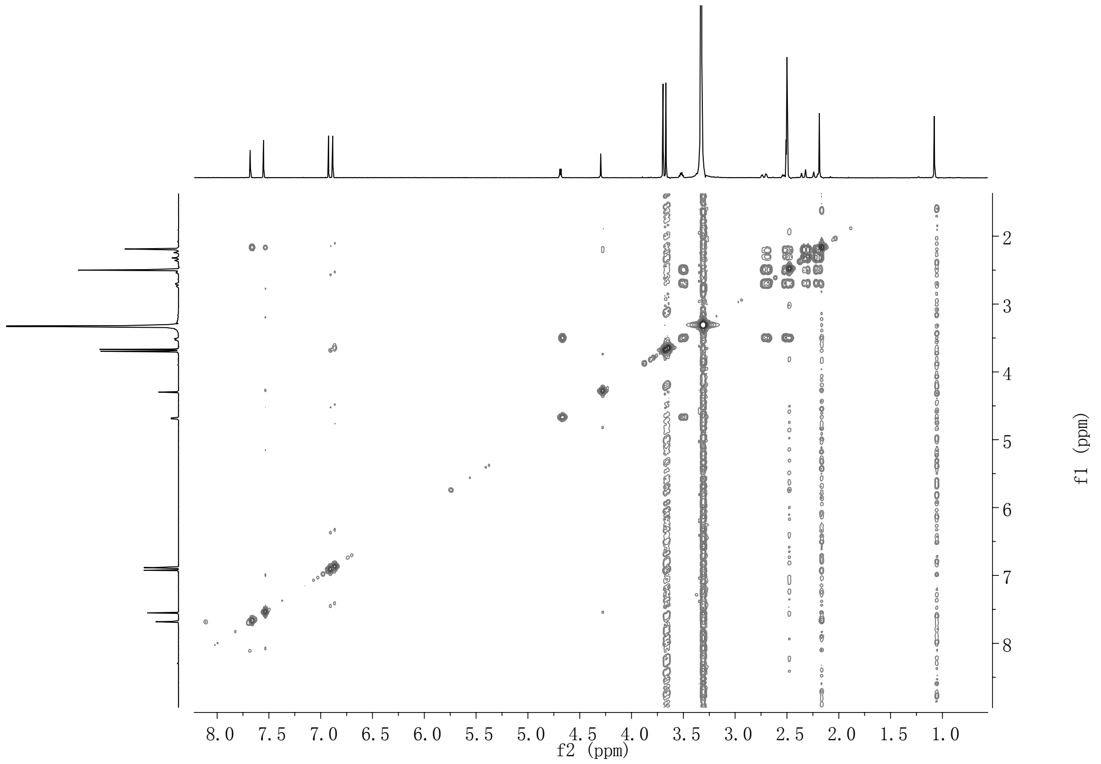

**Figure S7. HR-EIMS spectrum of 1.**

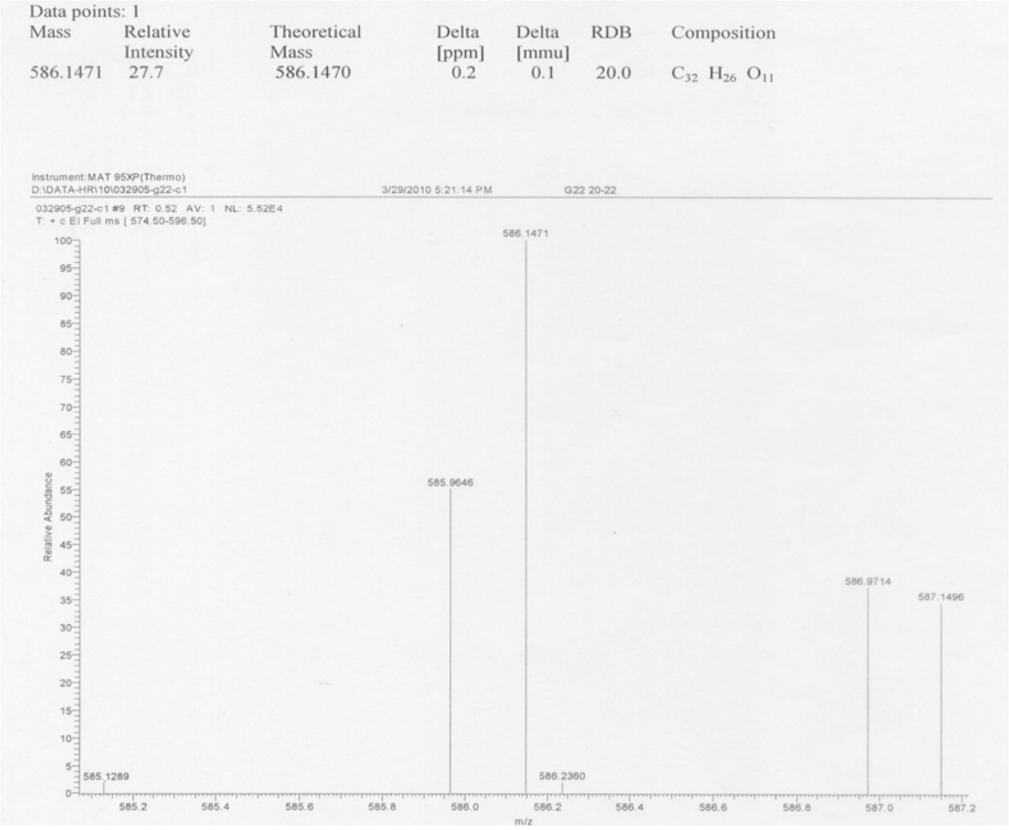


**Figure S8. $^{1}$H spectrum of 2 (**500 MHz, DMSO-$d_6$**).**

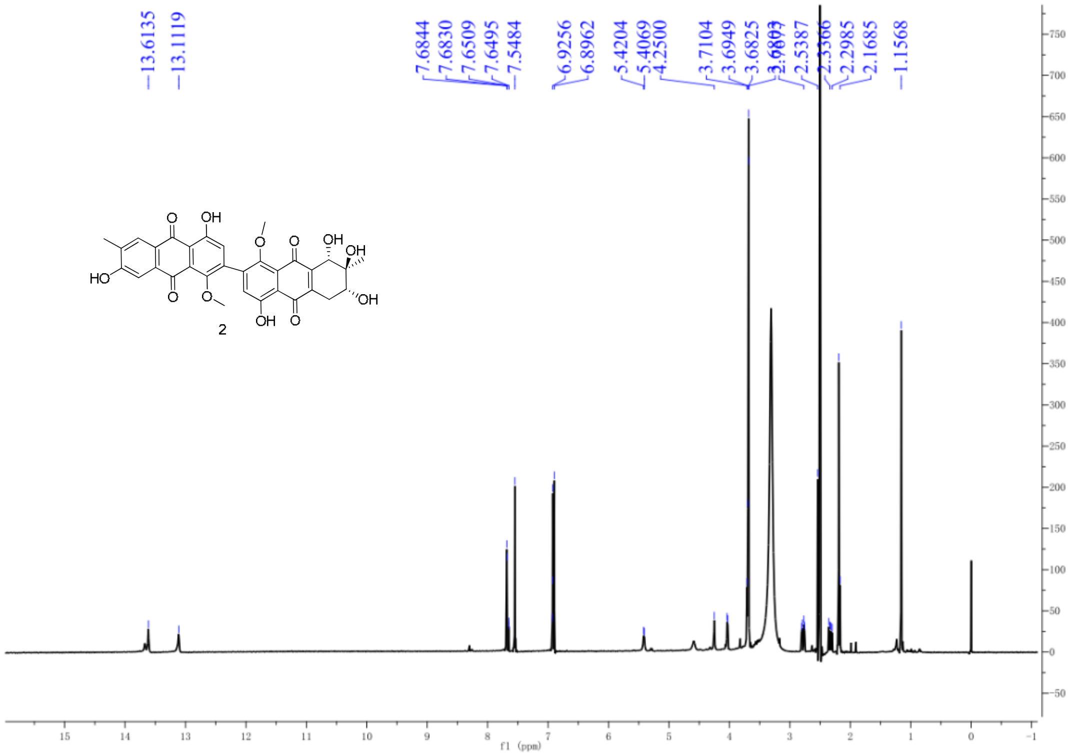

**Figure S9. $^{13}$C spectrum of 2 (**125 MHz, DMSO-$d_6$**).**

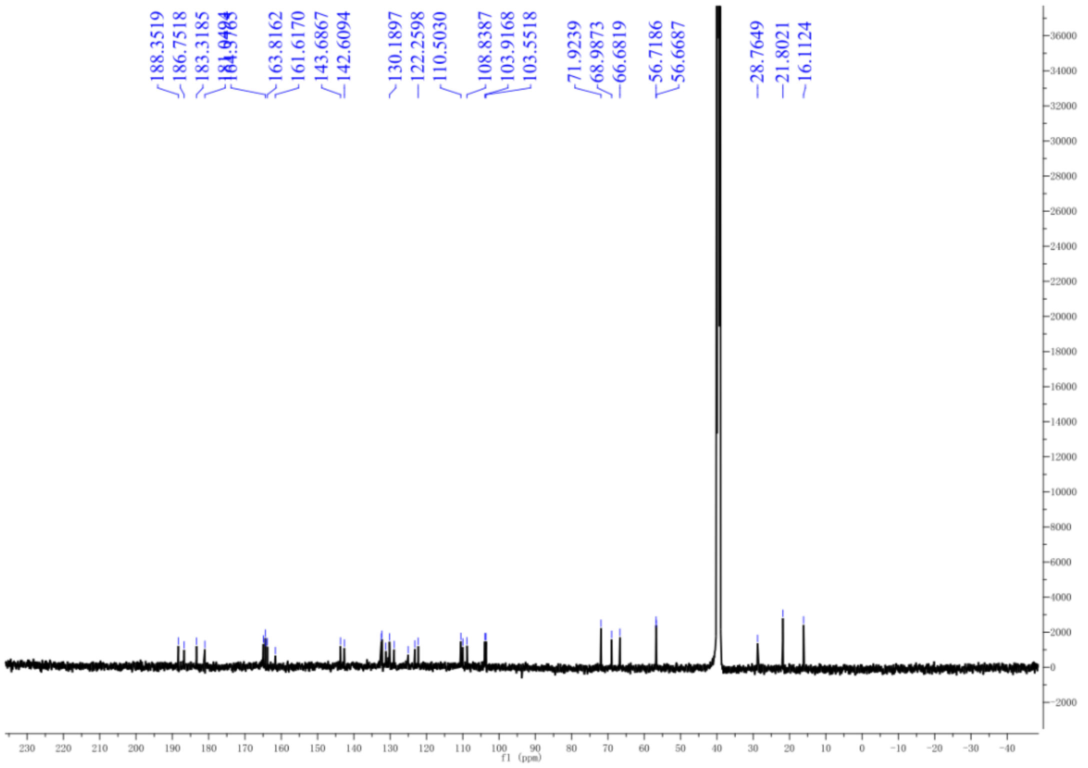


**Figure S10. HMQC spectrum of 2 (**500/125 MHz, DMSO-$d_6$**).**

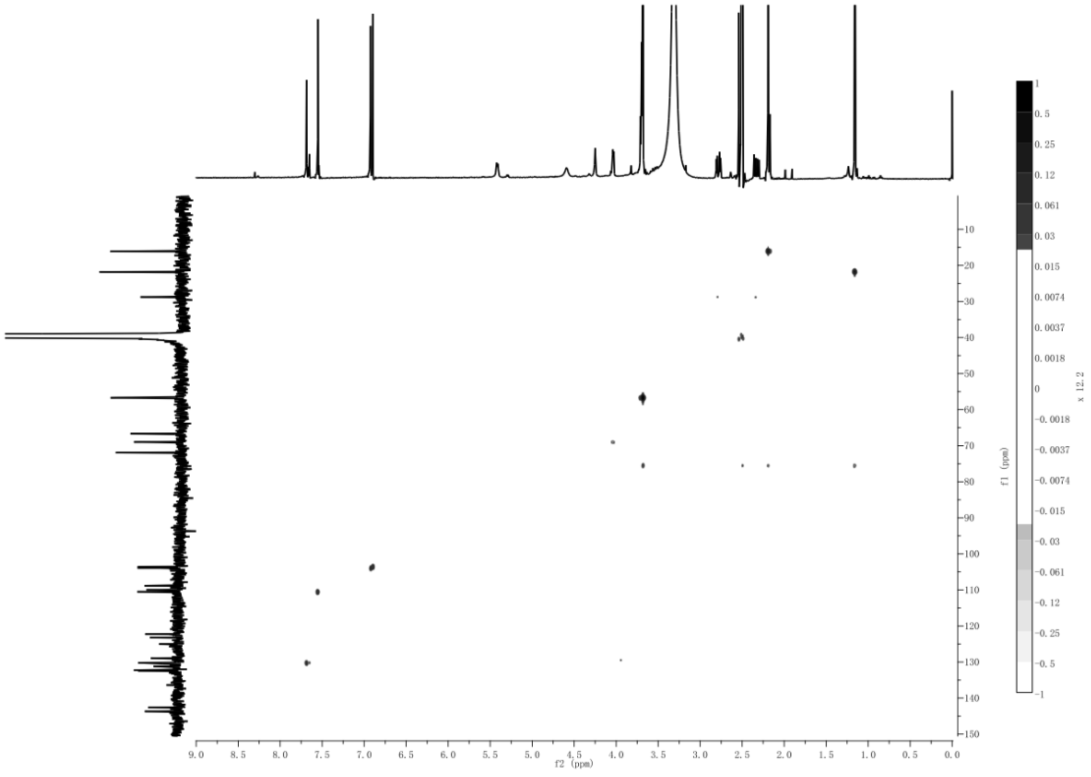

**Figure S11. HMBC spectrum of 2 (**500/125 MHz, DMSO-$d_6$**).**

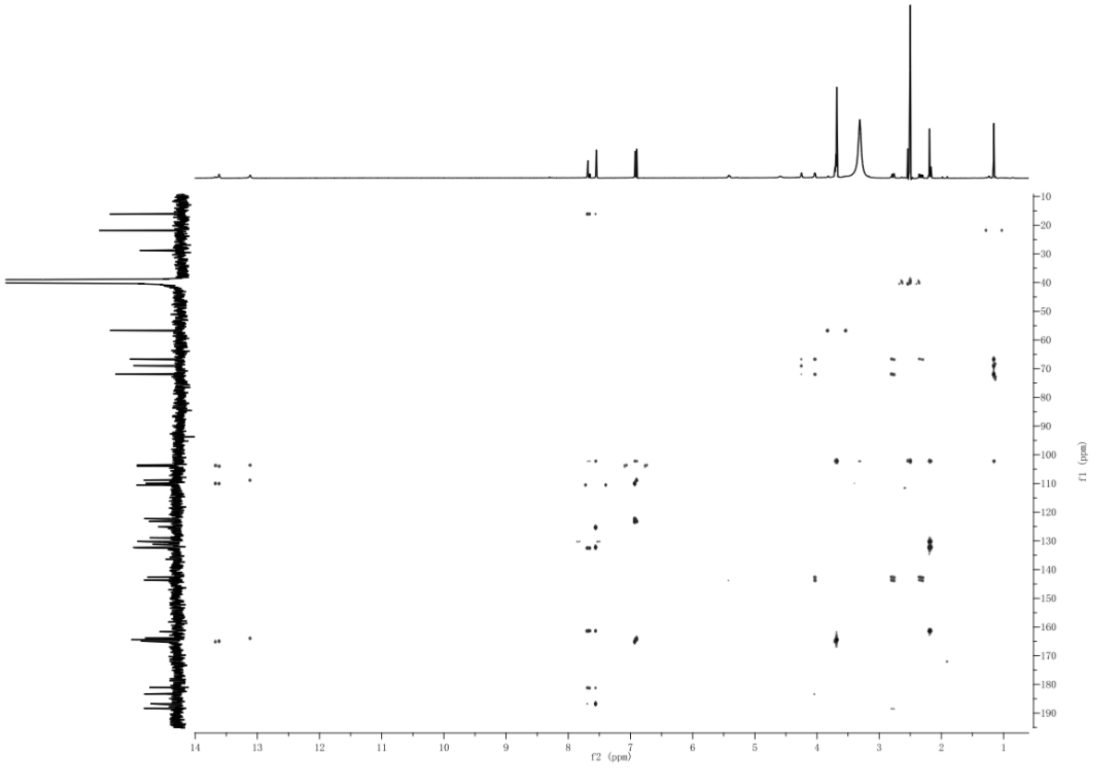


**Figure S12. $^1$H-$^1$H COSY spectrum of 2 (**500 MHz, DMSO-$d_6$**).**

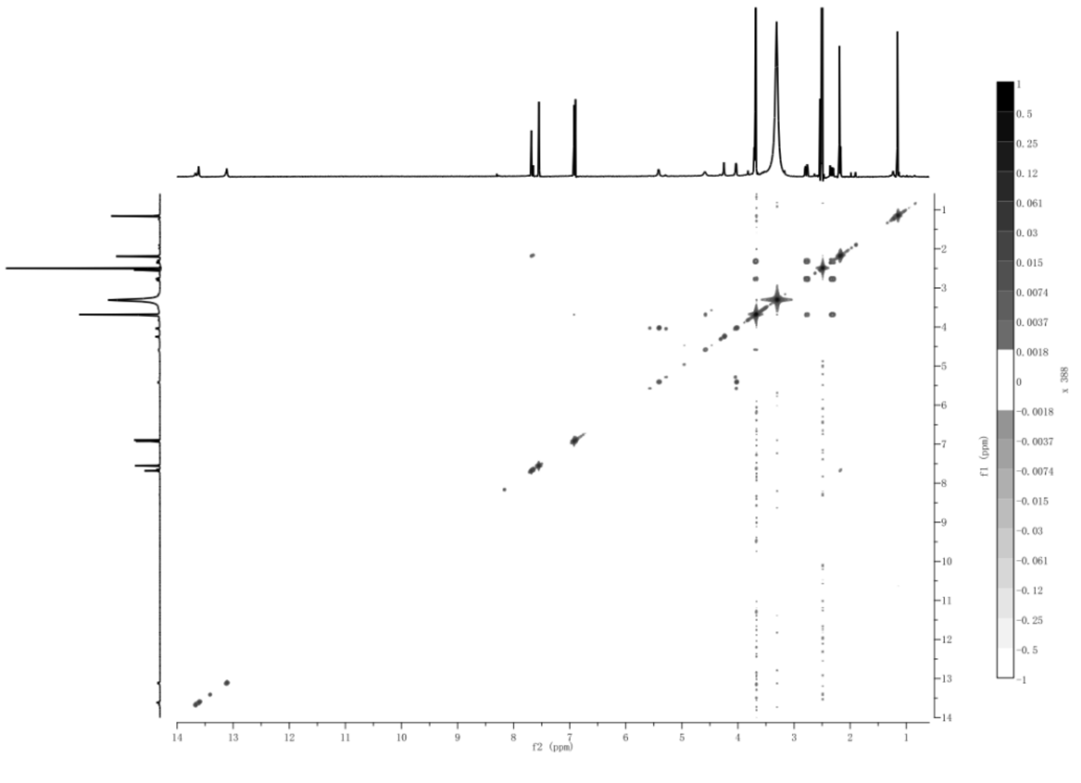

**Figure S13. HR-ESIMS spectrum of 2.**

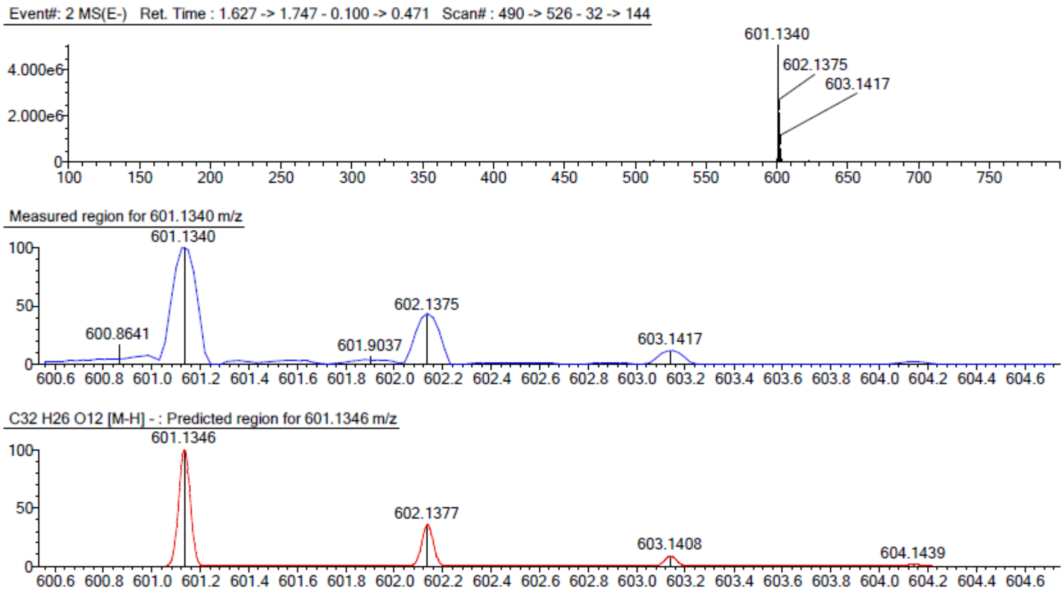


| Rank | Score | Formula (M) | Ion | Meas. m/z | Pred. m/z | Df. (mDa) | Df. (ppm) | Iso | DBE |
|---|---|---|---|---|---|---|---|---|---|
| 1 | 79.16 | C32 H26 O12 | [M-H] - | 601.1340 | 601.1346 | -0.6 | -1.00 | 79.16 | 20.0 |

**Figure S14. $^{1}$H spectrum of 3 (**500 MHz, DMSO-$d_6$**).**

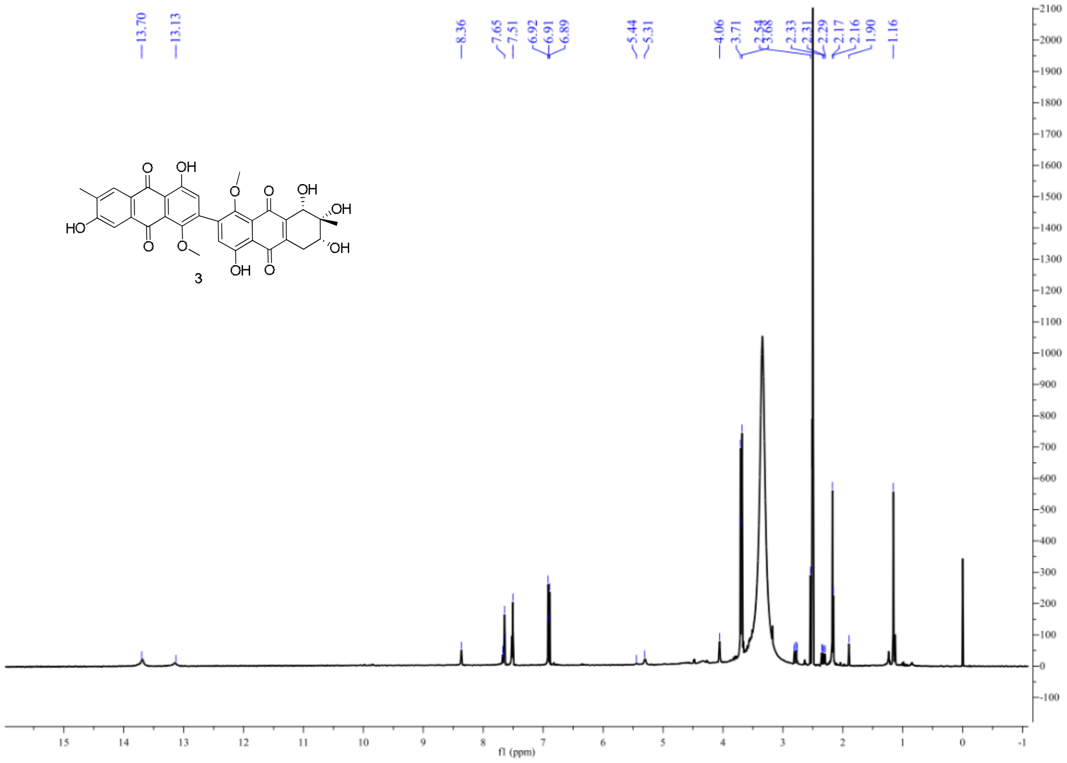

**Figure S15. $^{13}$C spectrum of 3 (**125 MHz, DMSO-$d_6$**).**

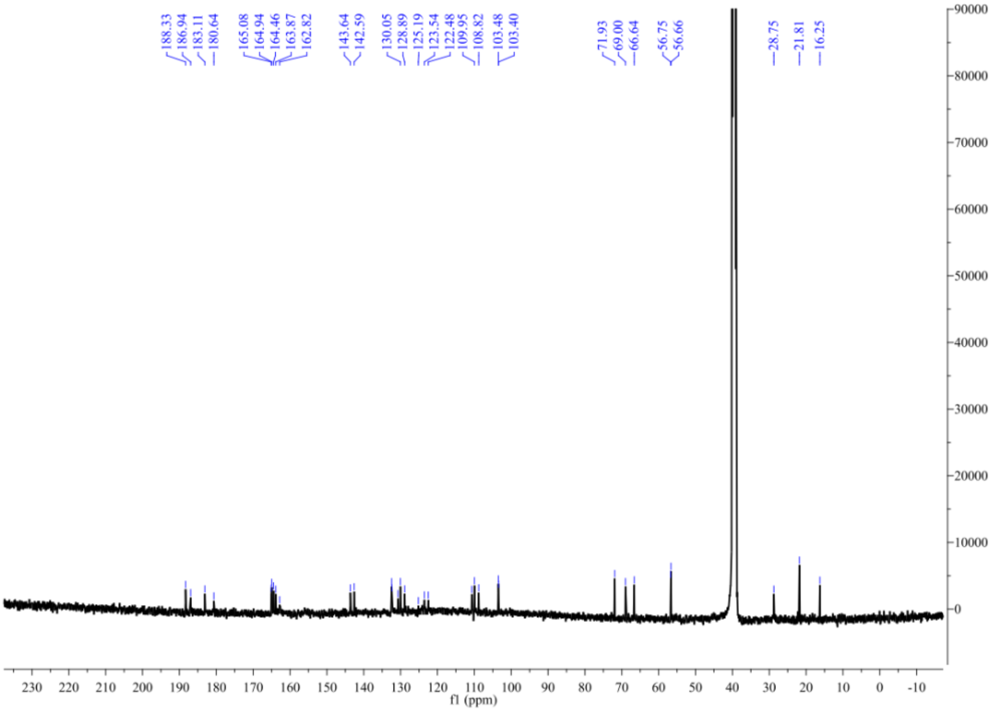


**Figure S16. HMQC spectrum of 3 (**500/125 MHz, DMSO-$d_6$**).**

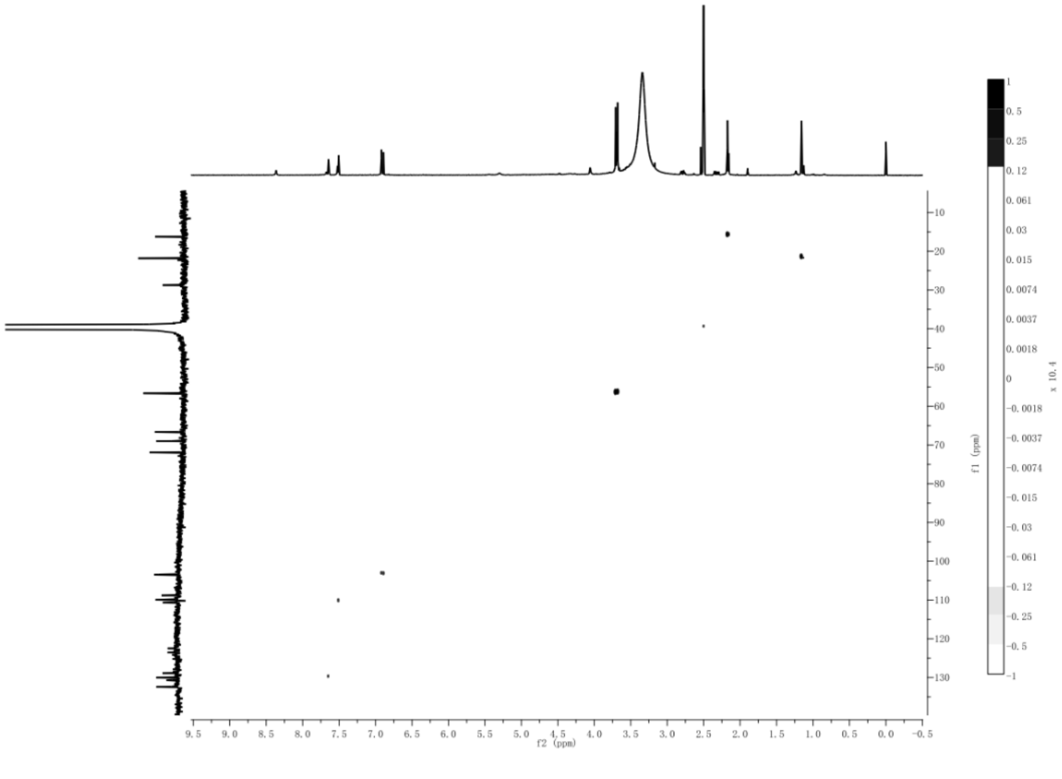

**Figure S17. HMBC spectrum of 3 (500/125 MHz, DMSO-$d_6$).**

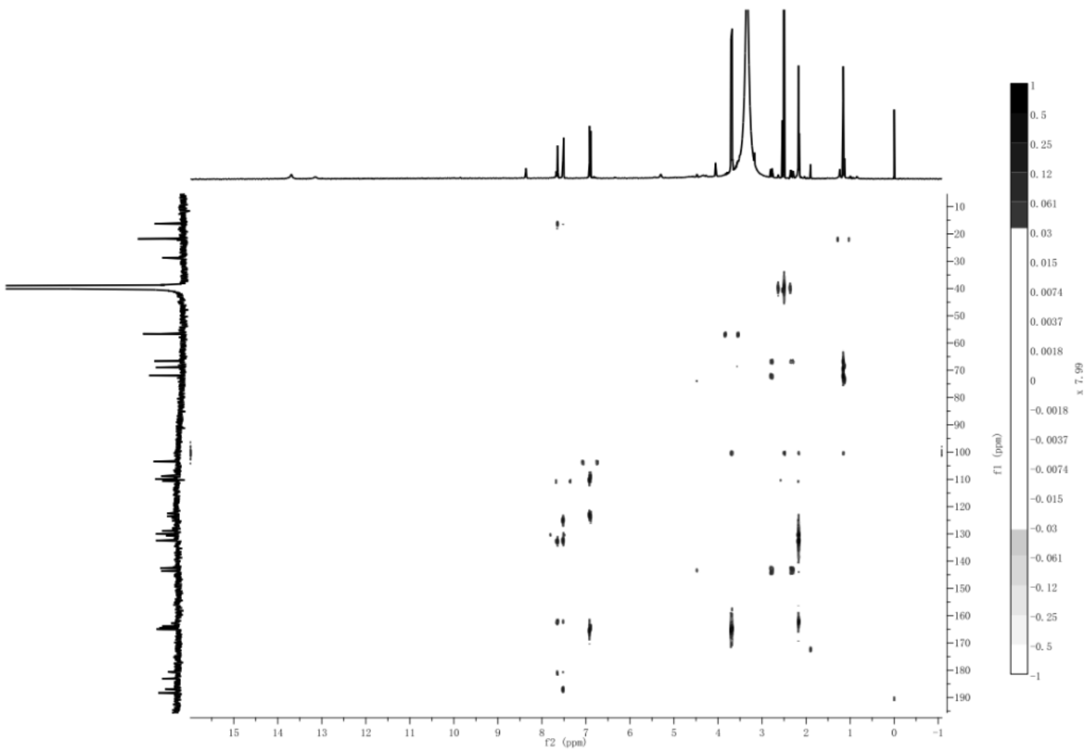


**Figure S18. $^1$H-$^1$H COSY spectrum of 3 (500 MHz, DMSO-$d_6$).**

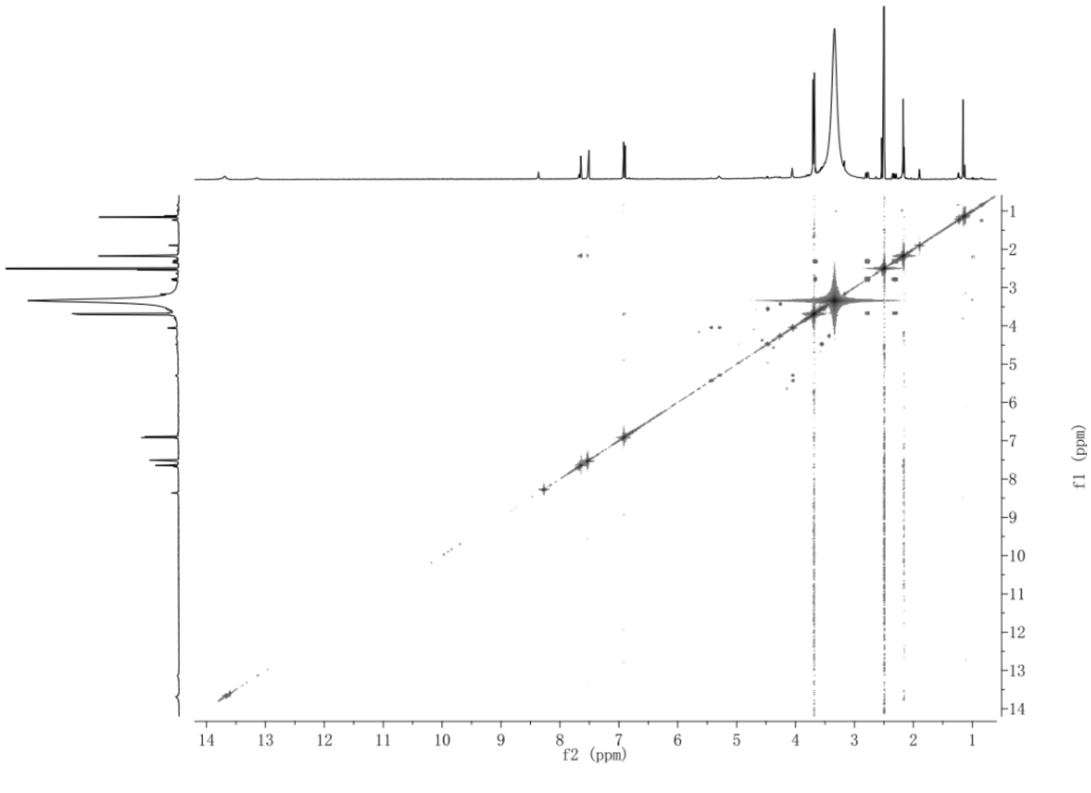

**Figure S19. HR-ESIMS spectrum of 3.**

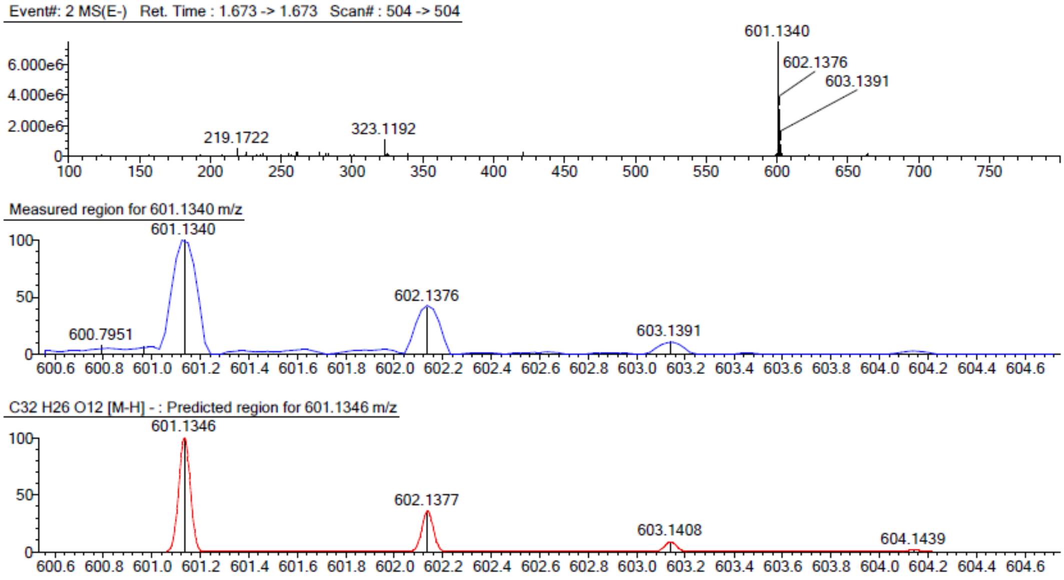


| Rank | Score | Formula (M) | Ion | Meas. m/z | Pred. m/z | Df. (mDa) | Df. (ppm) | Iso | DBE |
|---|---|---|---|---|---|---|---|---|---|
| 1 | 81.43 | C32 H26 O12 | [M-H] - | 601.1340 | 601.1346 | -0.6 | -1.00 | 81.43 | 20.0 |
